# Systematic Review and Meta-analysis of Chiropractic Care and Cervical Artery Dissection: No Evidence for Causation

**DOI:** 10.7759/cureus.498

**Published:** 2016-02-16

**Authors:** Ephraim W Church, Emily P Sieg, Omar Zalatimo, Namath S Hussain, Michael Glantz, Robert E Harbaugh

**Affiliations:** 1 Department of Neurosurgery, Penn State Hershey Medical Center

**Keywords:** vertebral atery dissection, cervical artery dissection, chiropractic manipulation, cervical manipulation, internal carotid artery dissection, cervical spine manipulative therapy

## Abstract

**Background:**

Case reports and case control studies have suggested an association between chiropractic neck manipulation and cervical artery dissection (CAD), but a causal relationship has not been established. We evaluated the evidence related to this topic by performing a systematic review and meta-analysis of published data on chiropractic manipulation and CAD.

**Methods:**

Search terms were entered into standard search engines in a systematic fashion. The articles were reviewed by study authors, graded independently for class of evidence, and combined in a meta-analysis. The total body of evidence was evaluated according to GRADE criteria.

**Results:**

Our search yielded 253 articles. We identified two class II and four class III studies. There were no discrepancies among article ratings (i.e., kappa=1). The meta-analysis revealed a small association between chiropractic care and dissection (OR 1.74, 95% CI 1.26-2.41). The quality of the body of evidence according to GRADE criteria was “very low.”

**Conclusions:**

The quality of the published literature on the relationship between chiropractic manipulation and CAD is very low. Our analysis shows a small association between chiropractic neck manipulation and cervical artery dissection. This relationship may be explained by the high risk of bias and confounding in the available studies, and in particular by the known association of neck pain with CAD and with chiropractic manipulation. There is no convincing evidence to support a causal link between chiropractic manipulation and CAD. Belief in a causal link may have significant negative consequences such as numerous episodes of litigation.

## Introduction

Neck pain is a common complaint in physicians’ and chiropractors’ offices. Data from the Centers for Disease Control and from national surveys document 10.2 million ambulatory care visits for a neck problem in 2001 and 2002. By comparison, there were 11 million office-based visits for ischemic heart disease [1]. Many patients with neck pain seek chiropractic care and undergo cervical manipulation. As many as 12% of North Americans receive chiropractic care every year, and a majority of these are treated with spinal manipulation [2].

In contrast to the frequency of neck pain and chiropractic treatments, spontaneous cervical artery dissection (CAD) is rare. The annual incidence of internal carotid artery dissection has been estimated at 2.5–3 per 100,000 patients and that of vertebral artery dissection at 1–1.5 per 100,000 [3]. Stroke occurs in a small proportion of those with CAD, and its true incidence is difficult to estimate. Overall, dissection accounts for two percent of all ischemic strokes [4].

Case reports and case series of cervical dissection following manipulation have been published. Despite their rarity, these cases are frequently publicized for several reasons. Patients are often young and otherwise in good health. Dissection accounts for 10–25% of ischemic strokes in young and middle aged patients [4]. If dissection is caused by cervical manipulation it is potentially a preventable condition. Recent reports, including case control studies, have suggested an association between chiropractic neck manipulation and cervical dissection [5-10]. Notably, a recent study from the American Heart Association evaluated the available evidence and concluded such an association exists [11]. This report did not include a meta-analysis, nor did it seek to classify studies and grade the body of evidence. We sought to examine the strength of evidence related to this question by performing a systematic review, meta-analysis, and evaluation of the body of evidence as a whole.

## Materials and methods

Search terms “chiropract*,” “spinal manipulation,” “carotid artery dissection,” “vertebral artery dissection,” and “stroke” were included in the search. We used the Medline and Cochrane databases. We additionally reviewed references of key articles for completeness. A librarian with expertise in systematic review was consulted throughout the search process.

Two study authors independently reviewed all articles (EC, ES). They selected any applicable studies for evaluation based on pre-specified inclusion and exclusion criteria. We included only human trials examining patients with carotid or vertebrobasilar artery dissection and recent chiropractic neck manipulation. We excluded non-English language studies. The articles were independently graded using the classification of evidence scheme adopted by the American Academy of Neurology [12-14]. A third author (MG) arbitrated any discrepancies in the class-of-evidence ratings for the included studies.

Data from all class II and III studies were included in a meta-analysis. A second meta-analysis excluding class III studies was also performed. The inverse variance method and a fixed effects model were employed. Additionally, we report results using a variable effects model. The analyses were performed using RevMan 5.3 software from the Cochrane Informatics and Knowledge Management Department. We did not compose a protocol for our review, although PRISMA and MOOSE methodologies were used throughout [15-16].

We evaluated the total body of evidence for quality using the GRADE system [17-20]. A final GRADE designation was achieved by consensus after discussions involving all study authors as recommended by GRADE guidelines. This system is designed to assess the total body of evidence rather than individual studies. The criteria include study design, risk of bias, inconsistency, indirectness, imprecision, publication bias, effect size, dose response, and all plausible residual confounding. Four possible final designations are specified: high, moderate, low, and very low quality.

## Results

### Results of the systematic review

**Figure 1:**
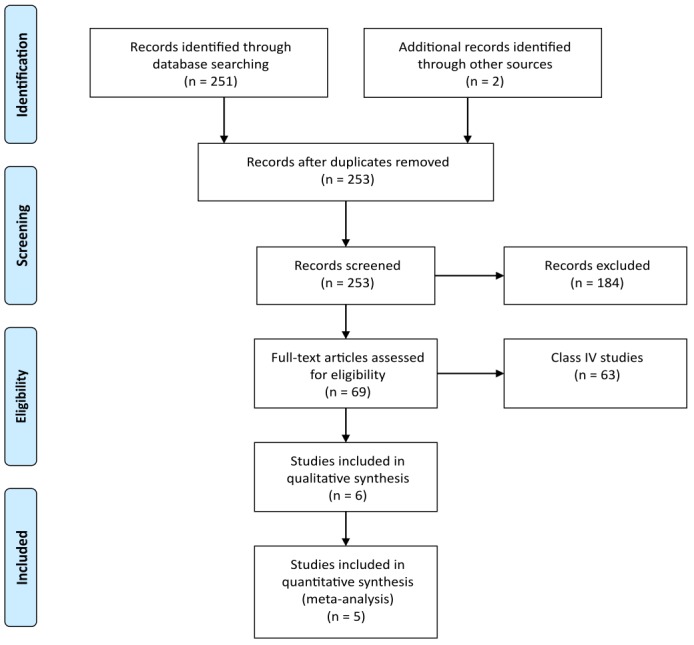
PRISMA flow diagram. [15]

Our search strategy yielded 253 articles. Seventy-seven were judged by all reviewers to be non-relevant. Four articles were judged to be class III studies, and two were rated class II. There were no discrepancies between the independent ratings (i.e., kappa=1). Studies rated class III or higher are listed in Table 1. Figure 1 outlines our process of selecting studies for inclusion in the meta-analysis.

**Table 1:** Class II and III articles identified in the systematic review. *Cases overlap with Cassidy et al., 2008. VBA = Vertebrobasilar accidents

Class II studies	Design	Patients	Number of dissections/VBA strokes
Smith et al., 2003	Case control	151	51
Dittrich et al., 2007	Case control	94	47
Class III studies			
Rothwell et al., 2001*	Case control	2910	582
Cassidy et al., 2008	Case control	3982	818
Thomas et al., 2011	Case control	90	47
Engelter et al., 2013	Case control	1897	966

### Meta-analysis

**Figure 2:**
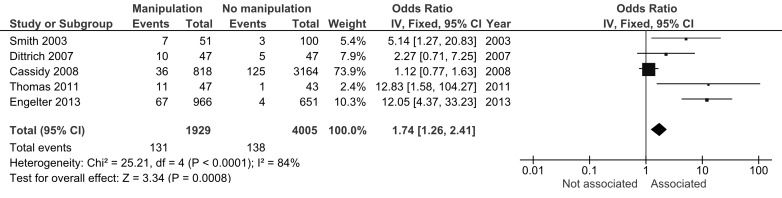
Meta-analysis of class II and III studies.

Combined data from class II and III studies suggests an association between dissection and chiropractic care, OR 1.74, 95% CI 1.26-2.41 (Figure 2). The result was similar using a random effects model, OR 4.05, 95% CI 1.27-12.91. We did not include the study by Rothwell et al. because it describes a subset of patients in the study by Cassidy et al. [5,8]. There was considerable heterogeneity among the studies (I^2^=84%).

We repeated the meta-analysis excluding class III studies. The combined effect size was again indicative of a small association between dissection and chiropractic care, OR 3.17, 95% CI 1.30-7.74). The result was identical when using a random effects model.

### Class II studies

Smith et al. used a retrospective case control design, combining databases from two academic stroke centers to identify cases of arterial dissection [9]. They found 51 cases and 100 controls. Exposure to spinal manipulative therapy (SMT) was assessed by mail survey. The authors reported an association between SMT and VBA (P = .032). In multivariate analysis, chiropractor care within 30 days was associated with VBA, even when adjusting for neck pain or headache (OR 6.6, 95% CI 1.4-30). While this study controlled for possible confounders such as neck pain, there were several limitations. Head and neck pain as well as chiropractor visit were assessed in a retrospective fashion by mail survey, very possibly introducing both recall and survivor bias. The reason for reporting to the chiropractor (e.g., trauma) was not assessed. Further, there was significant variability among diagnostic procedures, which may reflect increased motivation by physicians to rule out dissection in patients with a history of SMT. Such motivation could result in interviewer bias.

Dittrich et al. compared 47 patients with CAD to a control group with stroke due to etiologies other than dissection [6]. They assessed for risk factors using a face-to-face interview with blinding. These authors found no association between any individual risk factor and CAD, including cervical manipulative therapy. They blame the small sample size for the negative result, and they point out that cumulative analysis of all mechanical risk factors <24 hours prior to symptom onset showed an association (P = .01). This study is subject to recall bias.

### Class III studies

Rothwell et al. used a retrospective case control design to test for an association between chiropractic manipulation and vertebrobasilar accidents (VBA) [8]. They reviewed Ontario hospital records for admissions for VBA from 1993–1998. There were 582 cases and 2328 matching controls. The authors report an association between VBA and visit to a chiropractor within one week (OR 5.03, 95% CI 1.32-43.87), but this was only true for young patients (<45 years). This study represented the first attempt to delineate the association between chiropractic manipulation and extremely rare VBA with controls. Limitations included requisite use of *ICD-9 *codes to identify cases and associated classification bias, as well as potential unmeasured confounders (e.g., neck pain).

In 2008, Cassidy et al. set out to address the problem of neck pain possibly confounding the association between chiropractic care and VBA [5]. Again using a retrospective case control design, they included all residents of Ontario over a period of 9 years (1993–2002, 109,020,875 person years of observation). They identified 818 VBA strokes resulting in hospitalization and randomly selected age and sex matched controls. Next, they examined ambulatory encounters with chiropractors and primary care physicians (PCPs) in the one year preceding the stroke, limited to cervical manipulation, neck pain, and headache. Associations between chiropractor visit and VBA versus PCP visits and VBA were compared. Indeed, there were associations between both chiropractor visit and VBA (<45yrs OR 1.37, 95% CI 1.04-1.91), and PCP visit and VBA (<45 yrs OR 1.34, 95% CI .94-1.87; >45 yrs and OR 1.53, 95% CI 1.36-1.67). The association for chiropractor visit was not greater than for PCP visit. This data was interpreted as evidence that a confounder such as neck pain may account for the association between chiropractor visit and VBA. This study was subject to many of the same limitations as previous efforts. Canadian health records would not reveal whether a patient with cervical complaints underwent cervical manipulation, and the researchers could not review each chart for imaging confirming dissection. Additionally, the incidence of comorbidities (e.g., hypertension, heart disease, diabetes) was significantly higher among cases as compared to controls, and we are concerned that these differences were non-random.

In another case control study, Thomas et al. compared the records of 47 patients with confirmed or suspected vertebral or internal carotid artery dissection with 43 controls [10]. They limited their analysis to young patients defined as <55 years. These authors report a significant association between dissection and recent head or neck trauma (OR 23.51, 95% CI 5.71-96.89) as well as neck manual therapy (OR 1.67, 95% CI 1.43-112.0). An inconsistent standard for case ascertainment (a significant number of patients lacked radiographic confirmation of dissection) and lack of blinding weaken this study.

Engelter et al. evaluated data from the Cervical Artery Dissection and Ischemic Stroke Patients (CADISP) consortium, identifying 966 patients with CAD, 651 with stroke attributable to another cause, and 280 healthy controls [7]. The CADISP study involved both prospectively and retrospectively collected data at multiple centers in several countries. They assessed for prior cervical trauma within one month using questionnaires administered during clinic visits. Cervical manipulation therapy was more common for CAD versus stroke from another cause (OR 12.1, CI 4.37-33.2). The report notes that an association between any trauma and CAD was present even when restricting the analysis to prospectively recruited patients. However, in patients to whom the questionnaire was administered after dissection, recall bias may have been at work whether or not the patient was enrolled prospectively. Indeed, the frequency of prior cervical trauma in this study was substantially higher than previous reports (40% versus 12-34%). Additional weaknesses include a highly heterogeneous standard for case definition and no clear masking procedures.

### Body of evidence quality (GRADE rating)

Having performed a systematic review and rated articles according to their individual strengths and weaknesses, we graded the overall body of evidence using the system proposed by Guyatt et al. [17-20]. The GRADE approach to rating quality of evidence proposes four categories that are applied to a body of evidence: high, moderate, low, and very low. In the setting of systematic review, a particular rating reflects the extent of confidence that the estimates of effect are correct. The GRADE approach begins with study design and sequentially examines features with the potential to enhance or diminish confidence in the meta-analytic estimate of effect size.

Our final assessment of the quality of the body of evidence using these criteria was *very low.* The initial rating based on study design was *low* (observational studies). Given the controversial nature of this topic and the legal ramifications of results, there is certainly potential for bias (-1 serious). However, blinding in the Class II studies mitigated this risk to some extent. Inconsistency and imprecision did not lower our rating. Because the body of evidence is derived from measures of association, the rating was lowered for indirectness (-1 serious). Publication bias is less likely because of the impact of a negative result in this case. The funnel plot from our meta-analysis was inconclusive with regard to possible publication bias because of the small number of studies included but suggested a deficit in the publication of small negative trials. There was not a large effect size, and currently there is no evidence for a dose response gradient. Moreover, the most worrisome potential confounder (neck pain) would increase rather than reduce the hypothesized effect.

## Discussion

The results of our systematic review and meta-analysis suggest a small association between chiropractic care and CAD. There are no class I studies addressing this issue, and this conclusion is based on five class II and III studies. Scrutiny of the quality of the body of data using the GRADE criteria revealed that it fell within the “very low” category. We found no evidence for a causal link between chiropractic care and CAD. This is a significant finding because belief in a causal link is not uncommon, and such a belief may have significant adverse effects such as numerous episodes of litigation.

The studies included in our meta-analysis share several common weaknesses. Two of the five studies used health administrative databases, and since conclusions depend on accurate ICD coding, this technique for case ascertainment may introduce misclassification bias. It is not possible to account for the type of spinal manipulation that may have been used. Retrospective collection of data is also a potential weakness and may introduce recall bias when a survey or interview was used. Moreover, patients arriving at a hospital complaining of neck pain and describing a recent visit to a chiropractor may be subject to a more rigorous evaluation for CAD (interviewer bias). Another potential source of interviewer bias was lack of blinding in the class III studies. Further, we noted substantial variability among diagnostic procedures performed. All of these weaknesses affect the reliability of the available evidence and are not “corrected” by performing a meta-analysis.

**Figure 3:**
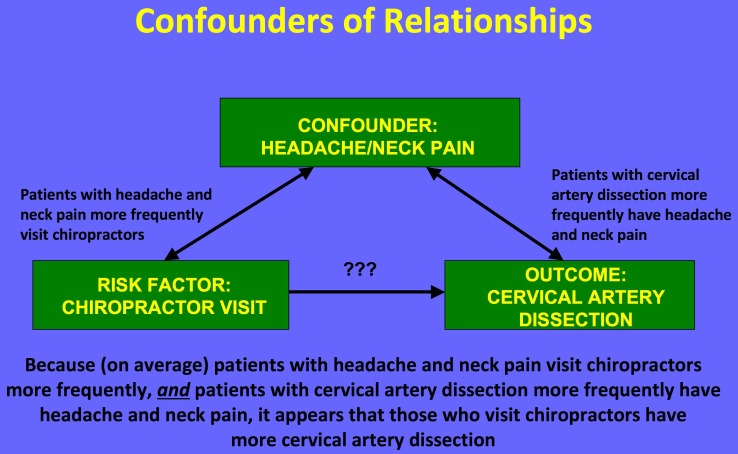
The association between a chiropractor visit and dissection may be explained by headache/neck pain, a likely confounder.

Perhaps the greatest threat to the reliability of any conclusions drawn from these data is that together they describe a correlation but not a causal relationship, and any unmeasured variable is a potential confounder. The most likely potential confounder in this case is *neck pain.* Patients with neck pain are more likely to have CAD (80% of patients with CAD report neck pain or headache) [21], and they are more likely to visit a chiropractor than patients without neck pain (Figure 3). Several of the studies identified in our systematic review provide suggestive evidence that neck pain is a confounder of the apparent association between chiropractic neck manipulation and CAD. For example, in Engelter et al. patients with CAD and prior cervical trauma (e.g., cervical manipulation therapy) were more likely to present with neck pain but less often with stroke than those with CAD and no prior cervical trauma (58% vs. 43% for trauma and 61% vs. 69% for stroke) [7]. If patients with CAD without neurological symptoms came to medical attention, it was probably because of pain. Patients with neck pain would also be more likely to visit a chiropractor than those without neck pain.

Cassidy et al. hypothesized that, although an association between chiropractor visits and vertebrobasilar artery stroke is present, it may be fully explained by neck pain and headache [5]. These authors reviewed 818 patients with vertebrobasilar artery strokes hospitalized in a population of 100 million person-years. They compared chiropractor and PCP visits in this population and reported no significant difference between these associations. For patients under 45 years of age, each chiropractor visit in the previous month increased the risk of stroke (OR 1.37, 95% CI 1.04-1.91), but each PCP visit in the previous month increased the risk in a nearly identical manner (<45 yrs OR 1.34, 95% CI .94-1.87; >45 yrs and OR 1.53, 95% CI 1.36-1.67). The authors conclude that, since patients with vertebrobasilar stroke were as likely to visit a PCP as they were to visit a chiropractor, these visits were likely due to pain from an existing dissection.

Cervical artery dissection is a rare event, creating a significant challenge for those who wish to understand it. A prospective, randomized study design is best suited to control for confounders, but given the infrequency of dissection, performing such a study would be logistically and also ethically challenging. Sir Austin Bradford Hill famously addressed the problem of assigning causation to an association with the application of nine tests [22]. These criteria include strength, consistency, specificity, temporality, biological gradient, plausibility, coherence, experimental evidence, and analogy. The specific tests and our assessment for the association between cervical manipulation and CAD are summarized in Table 2. In our appraisal, this association clearly passes only one test, it fails four, and the remaining four are equivocal due to absence of relevant data [23]. Further, a 2013 assessment of the quality of reports of cervical arterial dissection following cervical spinal manipulation similarly found lacking data to support a causal relationship [24]. 

**Table 2:** Hill’s criteria for assigning causation to association [22]. CAD = Cervical artery dissection

Criterion	Assessment for the Association Between Cervical Manipulation and CAD
1. Strength of the association	The association is modest.
2. Consistency of the association	Four of five class II and III studies demonstrate an association.
3. Outcome specific to exposure	As seen in Cassidy et al., exposure to a primary care doctor and exposure to a chiropractor are equally likely to result in CAD [5]. In this case the outcome is not specific.
4. Temporal relationship	A temporal relationship is necessary but not sufficient to establish causation. In this case, the onset of symptoms following cervical manipulation is variable and often delayed.
5. Biological gradient	There are no data to support or refute a dose-response hypothesis.
6. Plausibility	Are there increased rates of CAD in regions with increased utilization of chiropractic manipulation? No relevant data are available to address this criterion.
7. Coherence	Tests on human cadavers have revealed that vertebral artery strains during spinal manipulative therapy do not place significant strain on the vertebral artery [23]. A review of data related to this topic sponsored by the American Heart Association concluded that: “Current biomechanical evidence is insufficient to establish the claim that spinal manipulation causes [CAD], including data from a canine model showing no significant changes in [vertebral artery] lesions before and after cervical manipulation” [11].
8. Experimental evidence	The available animal models do not support the association.
9. Analogous to proven association	While severe trauma most certainly causes dissection, it may be debated whether the situation in chiropractic care is analogous.

In spite of the very weak data supporting an association between chiropractic neck manipulation and CAD, and even more modest data supporting a causal association, such a relationship is assumed by many clinicians. In fact, this idea seems to enjoy the status of medical dogma. Excellent peer reviewed publications frequently contain statements asserting a causal relationship between cervical manipulation and CAD [4,25,26]. We suggest that physicians should exercise caution in ascribing causation to associations in the absence of adequate and reliable data. Medical history offers many examples of relationships that were initially falsely assumed to be causal [27], and the relationship between CAD and chiropractic neck manipulation may need to be added to this list.

## Conclusions

Our systematic review revealed that the quality of the published literature on the relationship between chiropractic manipulation and CAD is very low. A meta-analysis of available data shows a small association between chiropractic neck manipulation and CAD. We uncovered evidence for considerable risk of bias and confounding in the available studies. In particular, the known association of neck pain both with cervical artery dissection and with chiropractic manipulation may explain the relationship between manipulation and CAD. There is no convincing evidence to support a causal link, and unfounded belief in causation may have dire consequences.

## References

[1] Riddle DL, Schappert SM (2007). Volume and characteristics of inpatient and ambulatory medical care for neck pain in the United States: data from three national surveys. Spine.

[2] Hurwitz EL, Chiang LM (2006). A comparative analysis of chiropractic and general practitioner patients in North America: findings from the joint Canada/United States survey of health, 2002-03. BMC Health Serv Res.

[3] Micheli S, Paciaroni M, Corea F (2010). Cervical artery dissection: emerging risk factors. Open Neurol J.

[4] Schievink WI (2001). Spontaneous dissection of the carotid and vertebral arteries. N Engl J Med.

[5] Cassidy JD, Boyle E, Côté PDC (2008). Risk of vertebrobasilar stroke and chiropractic care: results of a population-based case-control and case-crossover study. Spine.

[6] Dittrich R, Rohsbach D, Heidbreder A (2007). Mild mechanical traumas are possible risk factors for cervical artery dissection. Cerebrovasc Dis.

[7] Engelter ST, Grond-Ginsbach C, Metso TM (2013). Cervical artery dissection: trauma and other potential mechanical trigger events. Neurology.

[8] Rothwell DM, Bondy SJ, Williams JI (2001). Chiropractic manipulation and stroke: a population-based case-control study. Stroke.

[9] Smith WS, Johnston SC, Skalabrin EJ (2003). Spinal manipulative therapy is an independent risk factor for vertebral artery dissection. Neurology.

[10] Thomas LC, Rivett DA, Attia JR (2011). Risk factors and clinical features of craniocervical arterial dissection. Man Ther.

[11] Biller J, Sacco RL, Albuquerque FC (2014). Cervical arterial dissections and association with cervical manipulative therapy: a statement for healthcare professionals from the American Heart Association/American Stroke Association. Stroke.

[12] AAN (American Academy of Neurology) (2011). Neurology. Clinical Practice Guideline Process Manual. Clinical Practice Guideline Process Manual.

[13] French J, Gronseth G (2008). Lost in a jungle of evidence: we need a compass. Neurology.

[14] Gross RA, Johnston KC (2009). Levels of evidence: taking Neurology® to the next level. Neurology.

[15] Moher D, Liberati A, Tetzlaff J (2016). Preferred reporting items for systematic reviews and meta-analyses: the PRISMA statement. PLoS Med.

[16] Stroup DF, Berlin JA, Morton SC (2000). Meta-analysis of observational studies in epidemiology: a proposal for reporting. Meta-analysis of observational studies in epidemiology (MOOSE) group. JAMA.

[17] Guyatt G, Oxman AD, Akl EA (2011). GRADE guidelines: 1. Introduction-GRADE evidence profiles and summary of findings tables. J Clin Epidemiol.

[18] Guyatt GH, Oxman AD, Kunz R (2011). GRADE guidelines: 2. Framing the question and deciding on important outcomes. J Clin Epidemiol.

[19] Balshem H, Helfand M, Schünemann HJ (2011). GRADE guidelines: 3. Rating the quality of evidence. J Clin Epidemiol.

[20] The Cochrane Collaboration (2011). Reviews of Interventions (Version 5.1.0). Cochrane Handbook for Systematic Reviews of Interventions (Version 5.1.0).

[21] Lee VH, Brown RD Jr, Mandrekar JN (2006). Incidence and outcome of cervical artery dissection: a population-based study. Neurology.

[22] Hill AB (1965). The environment and disease: association or causation?. Proc R Soc Med.

[23] Herzog W, Leonard TR, Symons B (2012). Vertebral artery strains during high-speed, low amplitude cervical spinal manipulation. J Electromyogr Kinesiol.

[24] Wynd S, Estaway M, Vohra S, Kawchuk G (2016). The quality of reports on cervical arterial dissection following cervical spinal manipulation. PLOS ONE.

[25] Albuquerque FC, Hu YC, Dashti SR (2011). Craniocervical arterial dissections as sequelae of chiropractic manipulation: patterns of injury and management. J Neurosurg.

[26] Debette S, Leys D (2009). Cervical-artery dissections: predisposing factors, diagnosis, and outcome. Lancet Neurol.

[27] Artenstein AW (2012). The discovery of viruses: advancing science and medicine by challenging dogma. Int J Infect Dis.

